# Plum Pox Virus Strain C Isolates Can Reduce Sour Cherry Productivity

**DOI:** 10.3390/plants10112327

**Published:** 2021-10-28

**Authors:** Anna Sheveleva, Gennady Osipov, Tatiana Gasanova, Peter Ivanov, Sergei Chirkov

**Affiliations:** 1 Department of Virology, Faculty of Biology, Lomonosov Moscow State University, 119234 Moscow, Russia; anncsh@yandex.ru (A.S.); tv.gasanova@gmail.com (T.G.); regaflight@gmail.com (P.I.); 2 Tatar Research Institute of Agriculture, 420064 Kazan, Russia; osipovge@mail.ru

**Keywords:** *Prunus cerasus* L., sour cherry productivity, plum pox virus, cherry-adapted strains, ilarviruses

## Abstract

The impact of plum pox virus (PPV) on sour cherry (*Prunus cerasus* L.) productivity has been studied by comparing the yield of PPV-infected and PPV-free fruit-bearing trees. A total of 152 16- to 17-year-old trees of nine cultivars and hybrids were surveyed in the production orchards (cultivar collection and hybrid testing plots) in the Republic of Tatarstan, Russia. Sixty trees tested positive for PPV using ELISA and RT-PCR. Among them, 58 PPV isolates belonged to the strain C and the other 2 isolates to the strain CV. For the cultivars Sevastyanovskaya, Shakirovskaya, hybrids 88-2 and 80-8, the average (2012 to 2019) productivity of infected trees was 38% to 45% lower than for PPV-free trees of the same cultivar or hybrid. No ilarviruses (prunus necrotic ringspot virus, prune dwarf virus, apple mosaic virus, American plum line pattern virus) were detected in PPV-infected trees, suggesting that reduced cherry productivity was attributed to the PPV infection. Thus, it was shown for the first time that PPV can reduce the productivity of at least some sour cherry cultivars and hybrids, and strain C isolates are responsible for crop losses.

## 1. Introduction

Sour cherry (*Prunus cerasus* L.) and sweet cherry (*P. avium* L.) are economically important crops in many regions of the world, especially in Europe, the Mediterranean basin, the Middle East, and North America [1]. Over 30 viruses and viroids have been found on cherry [2,3,4]. Among them, ilarviruses from the genus *Ilarvirus* in the family *Bromoviridae* are prevalent viruses infecting sour and sweet cherry. The most common ilarviruses are prunus necrotic ringspot virus (PNRSV), prune dwarf virus (PDV), and apple mosaic virus (ApMV) [2,4,5,6]. The widespread nature of ilarviruses is due to their ability to be transmitted from plant to plant by pollen and through seeds at a high rate. Typical symptoms are chlorotic ringspots on leaves and fruits, yellow line patterns and rugose mosaics, although many trees often remain symptomless. Ilarviruses can cause significant yield loss, decreasing the growth of fruits and delaying fruit maturity [5]. 

Plum pox virus (PPV) is the etiological agent of sharka disease and the most devastating viral pathogen affecting stone fruit crops (*Prunus* spp.). Plum pox virus is a member of the genus *Potyvirus* in the family *Potyviridae*. It has a single-stranded positive-sense RNA genome of 9.8 kb. The large open reading frame (ORF) is translated into a 355 kDa polyprotein, which is processed by three virus-encoded proteases to ten mature proteins. A short overlapping ORF, called PIPO, is expressed as a P3N-PIPO fusion product essential for cell-to-cell virus movement [7], and the references therein]. Based on full-length genome analysis, ten PPV strains (D, M, Rec, EA, W, T, An, C, CR, CV) are recognized to date. Strains differ in antigenic and epidemiological properties, host preference and pathogenicity for different species and cultivars of stone fruit crops. Only strains C, CR and CV are able to infect sour and sweet cherries and are considered the cherry-adapted strains [8,9].

PPV-C seems to be the most widely distributed cherry-adapted strain. The monoclonal antibody AC and three sets of strain-specific primers, targeting the 3’-terminal genomic region, have been developed for its identification by ELISA and reverse transcription–polymerase chain reaction (RT-PCR) [10,11,12]. Using these detection tools, PPV-C isolates have been found sporadically on cherries in Moldova [13], Italy [14], Hungary [15], Belarus [16], Croatia [17], and Germany [18]. In contrast, PPV-C is widespread in Russia [19,20]. PPV-CR was discovered in Russia on sour cherry in the Middle Volga River basin in abandoned orchards, private gardens, and cultivar collections, as well as on wild sour cherry in Moscow [21,22]. PPV-CV was recently discovered on wild cherry in the Republic of Tatarstan [9]. RT-PCR assays with strain-specific primers were elaborated to identify these strains [9,21,22]. However, strains CR and CV have only been detected in Russia until now.

Typical symptoms of sharka disease are pale green rings, spots, and arabesques on leaves of infected plants, and they are rather similar in different *Prunus* species, including cherry. In plum (*P. domestica*), apricot (*P. armeniaca*), peach (*P. persica*), and myrobalan (*P. cerasifera*), PPV infection causes massive fruit drop and reduces fruit quality, making them unmarketable [7,23]. Due to sharka disease, annual losses in the main stone-fruit-growing regions are estimated at hundreds of millions of euros, and the number of PPV-infected trees destroyed since 1989 is in the millions [24]. In contrast, except for the leaf symptoms, the information on other manifestations of sharka disease in cherries is very scarce. Black ring patterns, depression, and necrosis, gradually disappearing during ripening, were observed on fruits of sour cherry infected with the PPV-C isolates SoC [10] and GC27 [18]. There are no data on the effect of the recently discovered CR and CV strains, since these isolates were mostly found in abandoned orchards and on wild, solitary, or other non-fruiting sour cherry trees. The economic significance of PPV for cherry remains questionable so far. 

Since PPV can cause significant crop losses in plum, peach, and apricot [7,23], we assumed that this virus should also affect cherry fruiting. The objectives of this work were to study the impact of PPV on sour cherry productivity by comparing the yield of PPV-infected and PPV-free trees, and to determine the strain of the virus isolates detected. It was shown for the first time that PPV can reduce the productivity of some cherry cultivars and hybrids. This effect was attributed to strain C isolates.

## 2. Results

**Figure 1:**
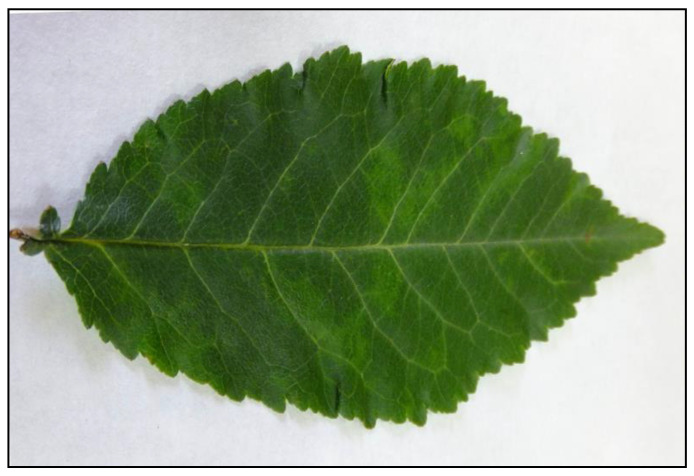
Symptoms on the sour cherry leaf infected with the plum pox virus isolate S9/32.

The study was performed at the Tatar zonal experimental station of horticulture (TZES) (N55.43; E49.01), Republic of Tatarstan, Russia. The productive sour cherry plantations (cultivar collection and hybrid orchards) cover 5.5 ha, which support 30 local cultivars and 500 hybrid forms (*n* = 2500 trees) (Figure S1). This location was chosen due to several reasons. First, a large number of sharka-affected trees was found in these plantings [20]. Leaf symptoms of sharka disease were first noticed in 2011. In 2012, all trees with conspicuous symptoms (Figure 1) were revealed and labeled as potentially PPV-infected. They were randomly distributed among the symptomless trees (data not shown). Second, multi-year data on each fruit-bearing sour cherry tree productivity have been gathered there, providing the yield of virus-infected and virus-free trees to compare.

To compare the yield of PPV-infected and PPV-free sour cherry plants, a total of 152 fruit-bearing trees of the cultivars Obilnaya, Zonalnaya, Sevastyanovskaya, Shakirovskaya, Nizhnekamskaya, and Truzhenitsa Tatarii, and hybrids 102-8, 88-2, and 80-8, were selected. Each of these cultivars and hybrids was represented by at least twelve 16- to 17-year-old trees that had from seven to ten skeletal branches. 

From 2018 to 2020, each of the selected trees were surveyed visually and tested for PPV using serological and molecular tests. Visual inspection revealed trees exhibiting leaf symptoms of sharka as well as symptomless ones. Using double-antibody sandwich ELISA (DAS-ELISA) and RT-PCR with P1/P2 primers, symptomatic leaves were shown to be PPV-infected, whereas all symptomless ones tested negative for the virus. The presence of PPV in the trees, which were marked in 2012 as potentially PPV-infected, was fully confirmed by both ELISA and RT-PCR. No newly infected trees among the aforementioned cultivars and hybrids were found. In sharka-affected trees, the symptoms were observed on one to ten skeletal branches. Trees on which at least one leaf displayed clear symptoms of sharka were considered PPV-infected. Eventually, PPV was found in 60 trees and the other 92 were PPV-free (Table 1). 

PPV was not detected in the cultivars Obilnaya, Zonalnaya, and 102-8 Hybrid. On the contrary, every Truzhenitsa Tatarii tree was infected with the virus. Among other cultivars and hybrids, only some trees were PPV-infected. For Sevastyanovskaya, Shakirovskaya, 88-2 and 80-8, the productivity of infected trees was 38% to 45% lower than that of PPV-free trees of the same cultivar or hybrid. The yield of infected and uninfected Nizhnekamskaya trees was similar. Thus, it was shown, for the first time, that PPV can reduce the productivity of at least some cherry cultivars and hybrids. Other manifestations of the disease, e.g., massive fruit drop or fruit deterioration typical of PPV-infected plum, apricot, and peach, have never been observed on PPV-infected cherries in Tatarstan. 

Even though no apparent symptoms of ilarviruses on leaves or fruits have been observed in the sour cherry plantings surveyed, PNRSV, PDV, ApMV, and American plum line pattern virus (APLPV) were tested using DAS-ELISA and RT-PCR in the PPV-infected samples, as ilarviruses can be latent [5]. No ilarviruses were detected in 60 PPV-infected trees (data not shown), suggesting that the reduced cherry productivity was attributed to the PPV infection. 

PPV isolates were further characterized by RT-PCR with the strain-specific primers developed for the identification of the strain C [11,12] and the strains D, M, W, CR, and CV [9,22,25,26]. The results are presented in Table S1. 

**Figure 2:**
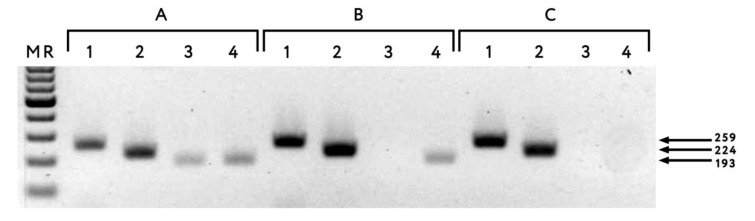
Agarose gel electrophoresis of RT-PCR products of ‘typical’ plum pox virus strain C isolate Ka34 (**A**) and SoC2-negative isolates NK11/16 (**B**) and Ka74 (**C**) generated with primers HSoC-1/CSoC-1 (lanes 1), M10-5’/M11-3’ (lanes 2), and HSoC-2/CSoC-2 (lanes 3–4). The primer annealing temperature was 62 °C (lanes 3) and 55 °C (lanes 4). MR GeneRuler 100 bpDNA ladder Plus (Thermo Scientific). The arrows on the right of the picture indicate the PCR products with sizes of 259, 224, and 193 base pairs.

From 60 isolates, 47 isolates were recognized by RT-PCR with the PPV-C-specific primer sets HSoC-1/CSoC-1, M10-5’/M11-3’, and HSoC-2/CSoC-2, generating the expected PCR products of 259, 224, and 193 base pairs (bp), respectively (Figure 2A, lanes 1–3). Eleven isolates tested negative with primers HSoC-2/CSoC-2, although these ‘SoC2-negative’ isolates were readily revealed using primers HSoC-1/CSoC-1 and M10-5’/M11-3’ (Figure 2B,C, lanes 1–3). Isolates of strains D, M, W, and CR were not detected in these samples, indicating no mixed infection with other strains of the virus. 

**Figure 3:**
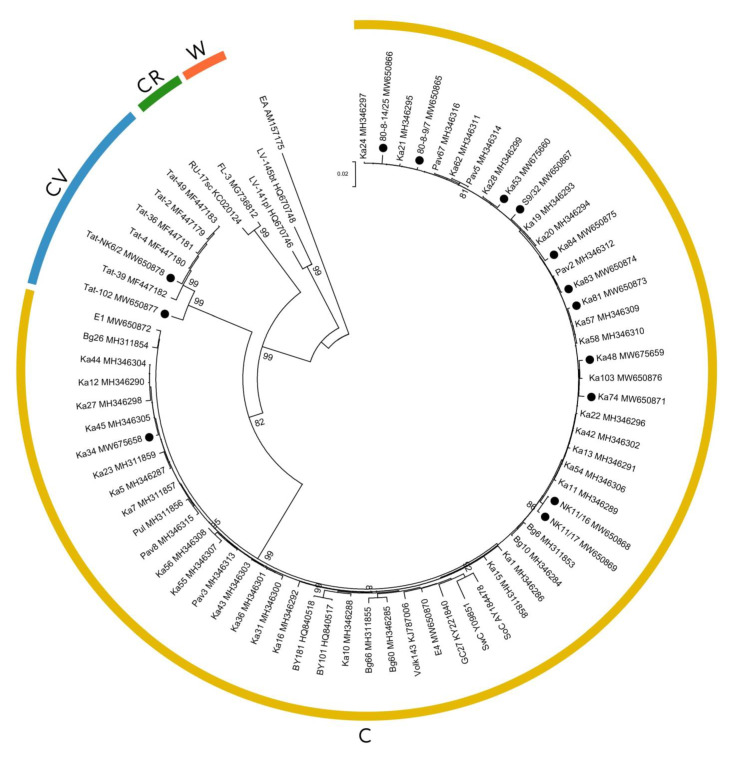
Phylogenetic analysis of coat protein gene sequences of all available plum pox virus isolates of the strains C and CV and selected isolates of the strains CR and W retrieved from GenBank. The tree was reconstructed using the neighbor joining algorithm and Kimura 2-paramenter model implemented in the MEGA7 program. Strain C, CV, CR, and W isolates are united with arcs in yellow, blue, green, and red, respectively. The isolate of the strain El Amar was used as the phylogenetic outgroup. Names of isolates and GenBank accession numbers are indicated at the end of branches. Bootstrap values (>80%) from 1000 replicates are shown next to the corresponding nodes. The isolates studied in this work are marked with a black circle (●). The scale bar indicates the number of substitutions per residue.

To further characterize the SoC2-negative isolates, the 3’-terminal regions of their genome encompassing the C-terminal segment of the *NIb* gene, entire coat protein (*CP*) gene, and 3’-untranslated region were sequenced and deposited in GenBank under accession numbers MW650865 to MW650869, MW650871, and MW650873 to MW650875. The corresponding 3’-terminal sequences of the SoC2-negative isolates Ka48 and Ka53 were retrieved from their full-size genomic sequences (MW675659, MW675660). According to BLASTn (https://blast.ncbi.nlm.nih.gov/Blast.cgi (accessed on 26 October 2021)), all of the sequences were most closely related to PPV-C isolate Ka7 (MH311857) from Tatarstan [20], sharing over 99% nucleotide (nt) identity. Similar to known cherry-adapted PPVs, the *CP* gene of the SoC2-negative isolates consisted of 996 nt, encoding a protein of 332 amino acids. Phylogenetic analysis of the *CP* gene of cherry-adapted isolates available in GenBank showed that the SoC2-negative isolates were assigned to the PPV-C clade (Figure 3). Based on the 3’-terminal sequence identity and phylogenetic analysis, all eleven SoC2-negative isolates undoubtedly belong to strain C. Thus, taking into account the results of RT-PCR, sequence identity, and phylogeny of the 3’-terminal genome regions, the vast majority of new PPV isolates (58 from 60) identified in this study belong to strain C. 

Analysis of the 3’-terminal genome segment of the SoC2-negative isolates revealed two or three nt mismatches between the HSoC-2/CSoC-2 primers and target *CP* gene (Table 2). Although minor, these differences could have affected the amplification of the PCR product due to the high annealing temperature of the primers (62 °C) [10]. Indeed, when the annealing temperature was lowered to 55 °C, the expected PCR product of 193 bp was generated for the isolates, showing two, but not three, mismatches (Figure 2B,C, lanes 4). No mismatches between two other PPV-C-specific primer sets and corresponding genome sequences of the SoC2-negative isolates were found (data not shown). To improve PPV-C detection using primers HSoC-2/CSoC-2, lowering the annealing temperature to 55 °C is recommended. 

Isolates Tat-NK6/2 and Tat-102, from the cultivars Nizhnekamskaya and Truzhenitsa Tatarii, respectively, were recognized by RT-PCR with PPV-CV- and PPV-D-specific primers, suggesting that they are the representatives of the strain CV [9]. The 3’-terminal genome regions of these isolates were sequenced and deposited in GenBank under accession numbers MW650877 and MW650878. Phylogenetic analysis of the *CP* gene confirmed their belonging to strain CV (Figure 3). Until recently, all PPV-CV isolates were only found on wild trees away from productive orchards [9]. In this work, isolates of the strain CV were detected for the first time in the functioning cultivar collection on fruit-bearing trees. The entering of PPV-CV to the productive orchard may result in the spreading of this rare strain to other cherry-growing regions through infected plant material.

Thus, PPV can reduce the productivity of at least some sour cherry cultivars and hybrids, and strain C isolates are mainly responsible for crop losses.

## 3. Discussion

The impact of PPV on sour cherry productivity has been studied. This work became possible because extensive sour cherry plantations of the TZES are massively infected with PPV and, at the same time, multi-year data on the productivity of each of the fruit-bearing trees have been collected. When comparing the yield of PPV-infected and PPV-free trees, it was found, for the first time, that PPV can reduce the sour cherry productivity.

The molecular characterization of the PPV isolates was an essential part of this work. It was shown that the decrease in cherry productivity was mainly due to isolates of strain C. However, other PPV strains can affect cherries in a different way. Thus, the average productivity of PPV-infected trees of the cultivars Nizhnekamskaya and Truzhenitsa Tatarii was 5.8 and 5.9 kg/tree, respectively (Table 1). At the same time, the productivity of trees of the same cultivars infected with the PPV-CV isolates (Tat-NK6/2 and Tat-102, Table S1) was below average and amounted to 3.2 and 4.6 kg/tree, respectively (data not shown).

It is worth noting that data on the productivity from 2012 to 2019 were taken into account, whereas PPV testing by ELISA and RT-PCR has only been carried out since 2018. However, symptoms of sharka disease on the trees, which were proven to be PPV-infected in this work using the laboratory tests, were observed at least since 2011, indicating that these trees were PPV-infected by the beginning of this study.

Ilarviruses were tested in PPV-infected samples as crop losses in mixed infections if detected could not be attributed to the sharka alone. In plum and peach, mixed infection with PPV, PNRSV, and PDV was shown to result in severe growth reduction, bark cancer, and the trees dying within a few years [23]. No ilarviruses were detected in the PPV-infected samples. Perhaps this result is not so surprising. Ilarviruses can indeed be widespread on sour cherry in some regions but this is not the case for others. For example, PNRSV and PDV were detected in 58.4% of sour cherry trees in the Iberian Peninsula [6], but large-scale surveys showed that their incidence in Bulgaria and Turkey does not exceed 15% [27]. Apparently, the reduced cherry productivity was attributed to PPV infection.

Whether this is due to a decrease in the size of fruits or their number, or both, remains to be determined. Interestingly, the yield of infected and uninfected Nizhnekamskaya trees was similar (Table 1). Perhaps this is due to the fact that only one or two skeletal branches of trees of this cultivar were PPV-infected. On the contrary, in other cultivars and hybrids, symptoms of sharka were observed on six to ten branches. The irregular distribution of PPV around the canopy is common for peach, apricot, and plum [7], and apparently for sour cherry, too. Alternatively, the cultivar Nizhnekamskaya may be tolerant to sharka.

PPV was not detected in the trees of the cultivars Obilnaya, Zonalnaya, and hybrid 102-8. It should be stressed that these trees grow up with other cultivars and hybrids, for many of which a high incidence of sharka disease was demonstrated. Thus, 35 cherry-adapted PPV isolates were previously found in the TZES [20], and 60 others were revealed in this work. The fact that Obilnaya, Zonalnaya, and 102-8 trees growing under high disease pressure, remain PPV-free over an extended period of time (16 to 17 years) may indicate their resistance to the virus.

## 4. Materials and Methods

### 4.1. Plants

A total of 152 fruit-bearing sour cherry trees of the cultivars Obilnaya, Zonalnaya, Sevastyanovskaya, Shakirovskaya, Nizhnekamskaya, and Truzhenitsa Tatarii, and hybrids 102-8, 88-2, and 80-8, were surveyed. Each of these cultivars and hybrids was represented by at least twelve 16- to 17-year-old trees that had from seven to ten skeletal branches. All of them were obtained by the vegetative propagation of one or a few mother plants of the corresponding cultivar or hybrid and grown from rooted cuttings without rootstock usage.

### 4.2. Sampling

The leaf samples were collected from late June to early July before harvest in 2018 to 2020. Four to five leaves from each tree were pooled into a composite sample for subsequent assays. In asymptomatic trees, mature leaves were collected from four to five branches around the canopy. In sharka-affected trees, leaves from symptomatic and symptomless branches were pooled and analyzed separately. Each of the 152 samples was tested for PPV and ilarviruses using two independent methods (ELISA and RT-PCR) according to the validated diagnostic protocol [28].

### 4.3. ELISA

DAS-ELISA was performed as described previously [20] using Agdia reagent sets for the detection of PPV (Cat. No. SRA 31505), American plum line pattern virus (APLPV) (SRA 32000), PDV (SRA 98700), and PNRSV (SRA 30500). Corresponding positive and negative controls for ilarviruses were purchased from Agdia. DAS-ELISA of ApMV was performed using the apple mosaic ilarvirus set (Loewe Biochemica GmbH, Sauerlach, Germany). The leaf tissue was ground in phosphate-buffered saline (PBS) supplemented with 0.02% (v/v) Tween 20, 2% (w/v) polyvinylpyrrolidone (MW of about 40,000), 0.5% (v/v) Triton X-100 and 0.02% (w/v) sodium azide using a 1/20 ratio (w/v). The clarified extracts were placed in the MaxiSorp microplate wells (Nunc) pre-coated with polyclonal virus-specific antibodies and incubated for 2 h at 37 °C. The subsequent steps were performed according to the kit/set manufacturer’s instructions. The optical densities were measured 40 min after substrate addition at the wavelength of 405 nm.

### 4.4. RT-PCR

Total RNA from sour cherry leaves was isolated using an RNeasy Plant Mini Kit (Qiagen) according to the manufacturer’s protocol. Random hexamer primers and Moloney murine leukemia virus (MMLV) reverse transcriptase (both from Evrogen, Moscow, Russia) were used for the first-strand cDNA synthesis. The RT products were employed in PCR for (i) PPV and ilarvirus detection using the generic primer sets P1/P2 [29] and Ilar2F5/Ilar2R9 [30], respectively, (ii) PPV strain identification, and (iii) sequencing of the 3’-terminal genome regions of PPV isolates. PCR products were analyzed by 2% (w/v) agarose gel electrophoresis and visualized by ethidium bromide staining.

### 4.5. Determination of PPV Strain

The PPV strain was identified by PCR using primer sets HSoC-1/CSoC-1, HSoC-2/CSoC-2, and M10-5’/M11-3’ developed for the strain C identification [11,12] as well as primers for strains D, M, W, CR, and CV identification, which were applied according to original protocols [9,22,25,26]. The isolates 1410 [31], H23, Pav17 [32], Fl-3 [21], Ka7 [20], and Tat-2 [9] from our laboratory collection were used as positive controls for the strains W, M, D, CR, C, and CV, respectively. PPV-free sour cherry leaves were employed as a negative control.

### 4.6. Sequencing of the 3’-Terminal Genomic Region

The 3’-terminal genome regions of PPV-C isolates spanning the entire *CP* gene and flanking sequences of the *NIb* gene and the 3′-untranslated region ((Cter)*NIb*-*CP*-3’-UTR) were amplified employing the forward primer p84, the reverse primer 4CPR1, and the proof-reading Encyclo DNA polymerase (Evrogen) [20]. PCR included denaturation at 94 °C for 30 s, primer annealing at 60 °C for 30 s, and extension at 72 °C for 1 min 40 s (35 cycles) with a final extension at 72 °C for 10 min. PCR products of about 1.2 kb were purified from agarose gel using BC022 Cleanup Standard kit (Evrogen) and sequenced in both directions by Evrogen. The (Cter)*NIb*-*CP*-3’-UTR sequences were assembled from overlapping PCR products. The 3’-terminal genome region of PPV-CV isolates was sequenced in the same way, with the exception that another CVseqNIbF forward primer, 5`-AAGCTGTACACCGATGAAGAGGCA-3`, and the annealing temperature of 56 °C were used [9]. The genome sequences were deposited in GenBank under accession numbers MW650865 to MW650869, MW650871, MW650873 to MW650875, MW650877, MW650878, and MW675658 to MW675660.

### 4.7. Sequence Analysis

Multiple alignments of genomic sequences using ClustalW, determination of their nucleotide identities, and phylogenetic analysis using the neighbor-joining algorithm and Kimura 2-parametric model were performed by the MEGA7 program [33]. Sequences of all available cherry-adapted PPV isolates were retrieved from GenBank and employed for the analyses.

### 4.8. Study on the Impact of PPV on Cherry Productivity

The yield was assessed by determining the weight of fruits collected from the entire tree. This operation was repeated annually for each fruit-bearing tree for many years. To determine the impact of PPV on cherry productivity, yield data for the trees of the aforementioned cultivars and hybrids (Section 4.1), collected between 2012 and 2019 were processed. The average yield of each of these trees for the specified period was calculated and matched with the data on their infestation with PPV. Based on these data, the average productivity of PPV-infected and PPV-free trees for each cultivar and hybrid for the specified period was calculated, and the data were expressed as means ± standard deviation (SD). The Student’s t-test was used for statistical analysis of differences between the average productivity of PPV-infected and PPV-free trees. The *p*-values less than 0.05 were considered statistically significant.

## 5. Conclusions

It has been shown, for the first time, that PPV can reduce the productivity of some sour cherry cultivars and hybrids. Other manifestations of the sharka disease, e.g., massive fruit drop or fruit deterioration common for PPV-infected plum, apricot, and peach have not been observed on PPV-infected cherries. No ilarviruses were detected in the PPV-infected samples, suggesting that the reduced cherry productivity was attributed to the PPV infection. The decrease in cherry productivity was mainly due to the PPV strain C isolates. Some of them were not recognized by the original RT-PCR assay using primers HSoC-2/CSoC-2 due to mismatches between primers and the target gene. Lowering the annealing temperature to 55 °C was recommended to improve PPV-C detection using primers HSoC-2/CSoC-2. PPV-CV isolates were detected for the first time in the productive sour cherry orchards, creating a threat of spreading the virus beyond the existing focus through infected plant material.

## Figures and Tables

**Table 1:** Productivity of sour cherry trees infected and uninfected with plum pox virus (PPV).

Cultivar/Hybrid	Number of Trees Infected/Tested with PPV	Fruit yield, kg/Tree ^a^		Yield Loss, %	*p*-Value ^b^
		Uninfected Trees	PPV-Infected Trees		
Obilnaya	0/17	7.7 ± 0.7	-	-	
Zonalnaya	0/13	6.8 ± 1.5	-	-	
Hybrid 102-8	0/13	8.1 ± 1.1	-	-	
Sevastyanovskaya	3/12	7.2 ± 1.1	4.2 ± 1.2	42	<0.05
Shakirovskaya	17/23	5.5 ± 0.7	3.4 ± 1.3	38	<0.01
Nizhnekamskaya	10/24	5.9 ± 1.4	5.8 ± 1.2	2	
Hybrid 88-2	5/12	4.7 ± 1.1	2.6 ± 1.4	45	<0.05
Hybrid 80-8	8/21	6.9 ± 1.4	4.0 ± 1.2	42	<0.01
Truzhenitsa Tatarii	17/17	-	5.9 ± 1.4	-	

^a^ Mean values ± SE over eight years (2012–2019), ^b^ Student’s t-test. “-”, no data.

**Table 2:** Alignment of PPV-C-specific primers HSoC-2/CSoC-2 and corresponding genome regions of SoC2-negative isolates.

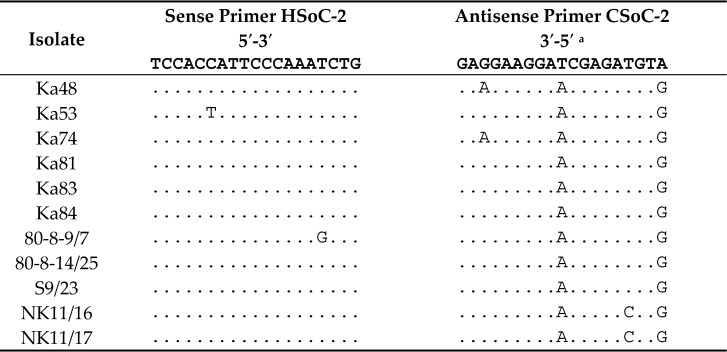

^a^ Reverse complement sequence of the primer (5′-TACATCTCGATCCTTCCTC-3′) is shown.

## Data Availability

The raw data that support the findings of this study (production per tree per year) are available from the corresponding author upon reasonable request. Sequencing data have been deposited in GenBank and their accession numbers are provided within the article.

## Supplementary Materials

The following are available online at https://www.mdpi.com/article/10.3390/plants10112327/s1, Figure S1: satellite map of sour cherry plantings surveyed in Tatarstan, Table S1: List of plum pox virus (PPV) isolates detected and studied in this work, Dataset 1: Genome sequences of PPV isolates deposited in GenBank.

## References

[1] Bujdoso G., Hrotko K., Quero-Garcia J., Iezzoni A., Pulawska J., Lang G. (2017). Cherry production. Cherries: Botany, Production and Uses.

[2] Myrta A., Savino V. (2008). Virus and virus-like diseases of cherry in the Mediterranean region. Acta Hortic..

[3] Rubio M., Martinez-Gomes P., Marais A., Sanchez-Navarro J.A., Pallas V., Candresse T. (2017). Recent advances and prospects in *Prunus* virology. Ann. Appl. Biol..

[4] James D., Cieslinska M., Pallas V., Flores R., Candresse T., Jelkmann W., Quero-Garcia J., Iezzoni A., Pulawska J., Lang G. (2017). Viruses, viroids, phytoplasmas and genetic disorders of cherry. Cherries: Botany, Production and Uses.

[5] Pallas V., Aparicio F., Herranz M.C., Amari K., Sanches-Pina M.A., Myrta A., Sanchez-Navarro J.A. (2012). Ilarviruses in *Prunus* spp.: A continued concern for fruit trees. Phytopathology.

[6] Perez-Sanchez R., Morales-Corts M.R., Gomes-Sanchez M.A. (2017). Sour and duke cherry viruses in South-West Europe. Phytopathol. Mediterr..

[7] Garcia J.A., Glasa M., Cambra M., Candresse T. (2014). *Plum pox virus* and sharka: A model potyvirus and a major disease. Mol. Plant Pathol..

[8] James D., Varga A., Sanderson D. (2013). Genetic diversity of Plum pox virus: Strains, diseases and related challenges for control. Can. J. Plant Pathol..

[9] Chirkov S., Sheveleva A., Ivanov P., Zakubanskiy A. (2018). Analysis of genetic diversity of Russian sour cherry *Plum pox virus* isolates provides evidence of a new strain. Plant Dis..

[10] Myrta A., Potere O., Crescenzi A., Nuzzaci M., Boscia D. (2000). Properties of two monoclonal antibodies specific to the cherry strain of *Plum pox virus*. J. Plant Pathol..

[11] Nemchinov L., Crescenzi A., Hadidi A., Piazzolla P., Verderevskaya T., Hadidi A., Khetarpal R.K., Kogazenawa H. (1998). Present status of the new cherry subgroup of plum pox virus (PPV-C). Plant Virus Disease Control.

[12] Szemes M., Kalman M., Myrta A., Boscia D., Nemeth M., Kolber M., Dorgai L. (2001). Integrated RT-PCR/nested PCR diagnosis for differentiating between subgroup of plum pox virus. J. Virol. Methods.

[13] Kalashyan J.A., Bilkej N.D., Verderevskaya T.D., Rubina E.V. (1994). Plum pox virus on sour cherry in Moldova. EPPO Bull..

[14] Crescenzi A., d’Aquino L., Comes S., Nuzzaci M., Piazzolla P., Boscia D., Hadidi A. (1997). Characterization of the sweet cherry isolate of plum pox potyvirus. Plant Dis..

[15] Nemchinov L., Hadidi A., Kolber M., Nemeth M. (2008). Molecular evidence for the occurrence of plum pox virus—Cherry subgroup in Hungary. Acta Hortic..

[16] Malinowski T., Sowik I., Salavei A.V., Kukharchyk N.V. Partial characterization of biological properties of PPV-C isolates found in Belarus and establishment of in vitro cultures of infected L2 and OWP-C rootstocks. Proceedings of the 22nd International Conference on Virus and Other Transmissible Diseases of Fruit Crops.

[17] Kajić V., Černi S., Škorić D. Plum pox virus on sour cherry in Croatia. Proceedings of the 22nd International Conference on Virus and Other Transmissible Diseases of Fruit Crops.

[18] Jelkmann W., Sanderson D., Berwarth C., James D. (2018). First detection and complete genome characterization of a Cherry (C) strain isolate of plum pox virus from sour cherry (*Prunus cerasus*) in Germany. J. Plant Dis. Prot..

[19] Glasa M., Shneyder Y., Predajňa L., Zhivaeva T., Prikhodko Y. (2014). Characterization of Russian Plum pox virus isolates provides further evidence of a low molecular heterogeneity within the PPV-C strain. J. Plant Pathol..

[20] Sheveleva A.A., Ivanov P., Gasanova T., Osipov G., Chirkov S. (2018). Sequence analysis of *Plum pox virus* strain C isolates from Russia revealed prevalence of the D96E mutation in the universal epitope and interstrain recombination events. Viruses.

[21] Chirkov S., Ivanov P., Sheveleva A. (2013). Detection and partial molecular characterization of atypical plum pox virus isolates from naturally infected sour cherry. Arch. Virol..

[22] Glasa M., Prikhodko Y., Predajna L., Nagyova A., Shneyder Y., Zhivaeva T., Subr Z., Cambra M., Candresse T. (2013). Characterization of sour cherry isolates of *Plum pox virus* from the Volga basin in Russia reveals a new cherry strain of the virus. Phytopathology.

[23] Nemeth M. (1994). History and importance of plum pox in stone-fruit production. EPPO Bull..

[24] Cambra M., Capote N., Myrta A., Llacer G. (2006). *Plum pox virus* and the estimated costs associated with sharka disease. EPPO Bull..

[25] Olmos A., Cambra M., Dasi M.A., Candresse T., Esteban O., Gorris M.T., Asensio M. (1997). Simultaneous detection and typing of plum pox potyvirus (PPV) isolates by hemi-nested PCR and PCR-ELISA. J. Virol. Methods.

[26] Glasa M., Malinowski T., Predajna L., Pupola N., Dekena D., Michalczuk L., Candresse T. (2011). Sequence variability, recombination analysis and specific detection of the W strain of *Plum pox virus*. Phytopathology.

[27] Kamenova I., Borisova A. (2021). Molecular variability of the coat protein gene of prunus necrotic ringspot virus on sweet and sour cherry in Bulgaria. J. Plant Pathol..

[28] IPPC-FAO. International Standards for Phytosanitary Measures: Diagnostic Protocols: *Plum pox virus*. ISPM 27, Annex 2 (DP2). 2018. https://assets.ippc.int/static/media/files/publication/en/2019/07/DP_02_2018_En_PlumPox_Rev_2018-09-21.pdf.

[29] Wetzel T., Candresse T., Macquaire G., Ravelonandro M., Dunez J. (1992). A highly sensitive immunocapture polymerase chain reaction method for plum pox potyvirus detection. J. Virol. Methods.

[30] Untiveros M., Perez-Egusquiza Z., Clover G. (2010). PCR assays for the detection of members of the genus *Ilarvirus* and family *Bromoviridae*. J. Virol. Methods.

[31] James D., Sanderson D., Varga A., Sheveleva A., Chirkov S. (2016). Genome sequence analysis of new isolates of the Winona strain of *Plum pox virus* and the first definitive evidence of intra-strain recombination events. Phytopathology.

[32] Sheveleva A.A., Mitrofanova I.V., Gorina V.M., Chirkov S.N. (2020). Molecular analysis of new Crimean isolates of the plum pox virus. Mosc. Univ. Biol. Sci. Bull..

[33] Kumar S., Stecher G., Tamura K. (2016). MEGA7: Molecular Evolutionary Genetics Analysis Version 7.0 for Bigger Datasets. Mol. Biol. Evol..

