# Supplementary material for: Plum Pox Virus Strain C Isolates Can Reduce Sour Cherry Productivity
Source: Plants (Basel). 2021 Oct 28;10(11):2327. doi: 10.3390/plants10112327 (PMC8621038; doi:10.3390/plants10112327)
Supplement: Supplementary file 1 [file plants-10-02327-s001.zip › Table S1.pdf]

Table S1. List of plum pox virus (PPV) isolates detected and studied in this work.

| **Cultivar/ Hybrid** | **PPV isolate**[a] | **Recognition by RT-PCR using PPV-C-specific primers**[b] | | | **PPV strain** | **Genomic region sequenced**[c] | **GenBank accession number** |
|---|---|---|---|---|---|---|---|
| | | HSoC-1/CSoc-1 | HSoC-2/CSoC-2 | M10-5'/M11-3' | | | |
| Nizhnekamskaya | NK5/3 | + | + | + | C | ns | |
| | Tat-NK6/2 | - | - | - | CV | CP | MW650878 |
| | NK7/5 | + | + | + | C | ns | |
| | NK7/7 | + | + | + | C | ns | |
| | NK7/8 | + | + | + | C | ns | |
| | NK7/9 | + | + | + | C | ns | |
| | NK11/12 | + | + | + | C | ns | |
| | NK11/14 | + | + | + | C | ns | |
| | NK11/16 | + | - | + | C | (Cter)NIb-CP-3'-UTR | MW650868 |
| | NK11/17 | + | - | + | C | (Cter)NIb-CP-3'-UTR | MW650869 |
| Sevastyanovskaya | S7/18 | + | + | + | C | ns | |
| | S7/23 | + | + | + | C | ns | |
| | S9/32 | + | - | + | C | (Cter)NIb-CP-3'-UTR | MW650867 |
| Shakirovskaya | SH1/24 | + | + | + | C | ns | |
| | Ka34 | + | + | + | C | Complete genome | MW675658 |
| | SH2/18 | + | + | + | C | ns | |

| | | | | | | | |
|---|---|---|---|---|---|---|---|
| | SH2/19 | + | + | + | C | ns | |
| | SH2/20 | + | + | + | C | ns | |
| | SH2/21 | + | + | + | C | ns | |
| | SH2/22 | + | + | + | C | ns | |
| | Ka81 | + | - | + | C | (Cter)NIb-CP-3'-UTR | MW650873 |
| | SH2/26 | + | + | + | C | ns | |
| | SH4/10 | + | + | + | C | ns | |
| | SH10/39 | + | + | + | C | ns | |
| | SH11/34 | + | + | + | C | ns | |
| | Ka83 | + | - | + | C | (Cter)NIb-CP-3'-UTR | MW650874 |
| | Ka84 | + | - | + | C | (Cter)NIb-CP-3'-UTR | MW650875 |
| | SH13/4 | + | + | + | C | ns | |
| | SH13/7 | + | + | + | C | ns | |
| | SH13/8 | + | + | + | C | ns | |
| Truzhenitsa Tatarii | Ka48 | + | - | + | C | Complete genome | MW675659 |
| | Ka74 | + | - | + | C | (Cter)NIb-CP-3'-UTR | MW650871 |
| | TT3/13 | + | + | + | C | ns | |

| | | | | | | | |
|---|---|---|---|---|---|---|---|
| | TT3/14 | + | + | + | C | ns | |
| | TT3/15 | + | + | + | C | ns | |
| | Tat-102 | - | - | - | CV | CP | MW650877 |
| | TT3/17 | + | + | + | C | ns | |
| | TT3/25 | + | + | + | C | ns | |
| | TT4/29 | + | + | + | C | ns | |
| | TT4/31 | + | + | + | C | ns | |
| | TT4/33 | + | + | + | C | ns | |
| | TT14/3 | + | + | + | C | ns | |
| | TT14/4 | + | + | + | C | ns | |
| | TT14/5 | + | + | + | C | ns | |
| | TT14/6 | + | + | + | C | ns | |
| | TT14/8 | + | + | + | C | ns | |
| | TT14/9 | + | + | + | C | ns | |
| 80-8 | 8/10 | + | + | + | C | ns | |
| | 8/12 | + | + | + | C | ns | |
| | 8/17 | + | + | + | C | ns | |
| | 9/4 | + | + | + | C | ns | |
| | 9/5 | + | + | + | C | ns | |
| | 80-8-9/7 | + | - | + | C | (Cter)NIb-CP-3'-UTR | MW650865 |

| | | | | | | | |
|---|---|---|---|---|---|---|---|
| | 14/18 | + | + | + | C | ns | |
| | 80-8-14/25 | + | - | + | C | (Cter)NIb-CP-3'-UTR | MW650866 |
| 88-2 | Ka53 | + | - | + | C | Complete genome | MW675660 |
| | 4/3 | + | + | + | C | ns | |
| | 4/5 | + | + | + | C | ns | |
| | 4/7 | + | + | + | C | ns | |
| | 14/11 | + | + | + | C | ns | |

[a] PPV-C SoC2-negative isolates are in yellow; PPV-CV isolates are in blue.

[b] "+" PCR product of the expected size (259, 193, and 224 bp, respectively); "-" no PCR product.

[c] CP - coat protein gene; (Cter)NIb-CP-3'-UTR - C-terminal segment of the NIb gene, entire coat protein gene and 3'- untranslated region of the genome.

ns - not sequenced.
