# Supplementary figures and images for: Plum Pox Virus Strain C Isolates Can Reduce Sour Cherry Productivity
Source: Plants (Basel). 2021 Oct 28;10(11):2327. doi: 10.3390/plants10112327 (PMC8621038; doi:10.3390/plants10112327)

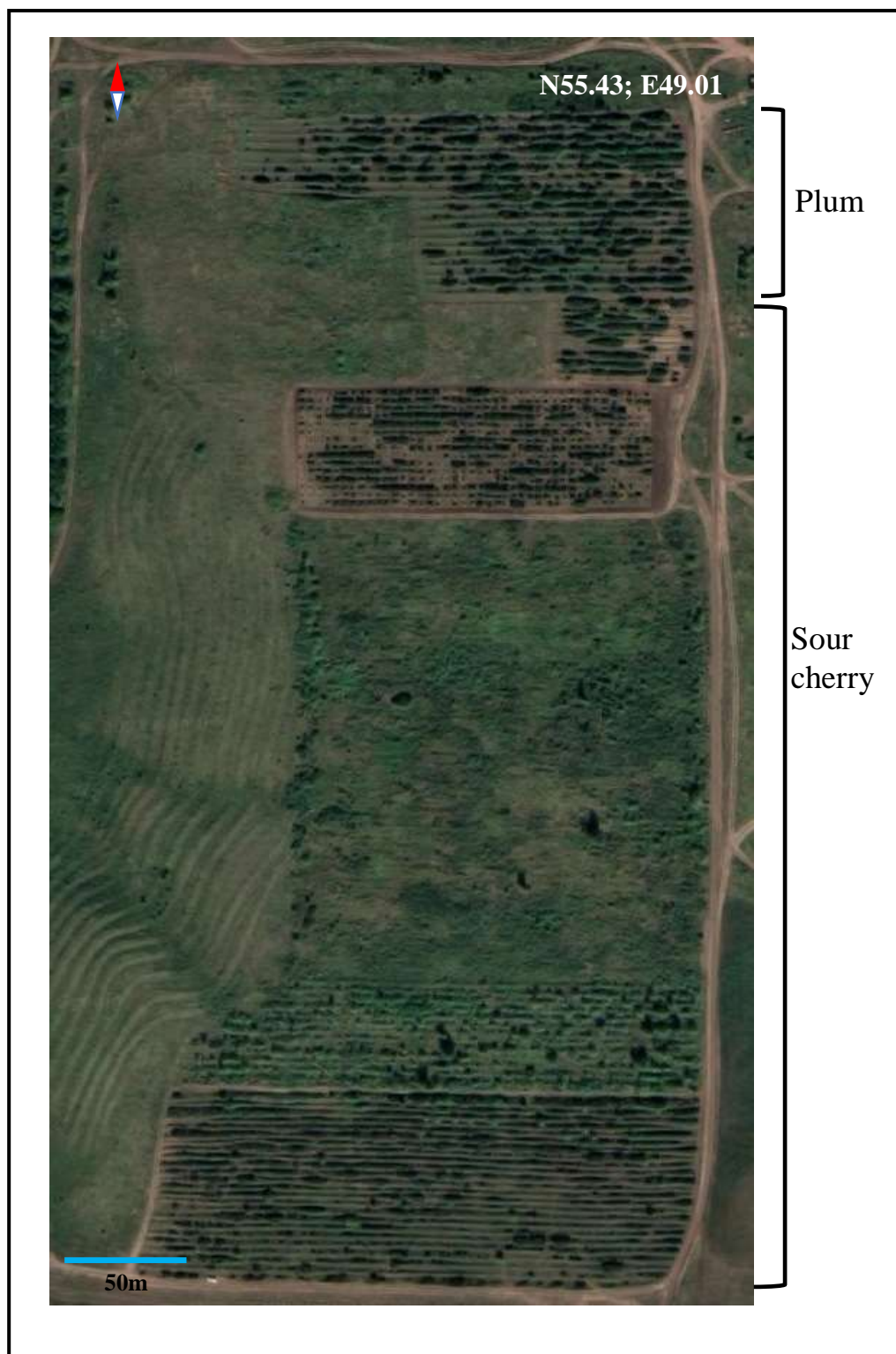

Supplement: Supplementary file 1 [file plants-10-02327-s001.zip › Figure S1.pdf]
